# Ultrasound Diagnosis of Pelvic Organ Prolapse Using Artificial Intelligence

**DOI:** 10.3390/jcm14113634

**Published:** 2025-05-22

**Authors:** José Antonio García-Mejido, Juan Galán-Paez, David Solis-Martín, Fernando Fernández-Palacín, Ana Fernández-Palacín, José Antonio Sainz-Bueno

**Affiliations:** 1 Department of Surgery, Faculty of Medicine, University of Seville, 41009 Seville, Spain; jsainz@us.es; 2 Department of Computer Science and Artificial Intelligence, Faculty of Computer Engineering, University of Seville, 41080 Seville, Spain; juangalan@us.es (J.G.-P.);; 3 Department of Statistics and Operational Research, University of Cadiz, 11510 Cadiz, Spain; fernando.fernandez@uca.es; 4 Biostatistics Unit, Department of Preventive Medicine and Public Health, University of Seville, 41009 Seville, Spain; afp@us.es

**Keywords:** machine learning, pelvic floor, ultrasonography, gradient boosting, XGBoost, artificial intelligence, pelvic organ prolapse

## Abstract

**Background/Objectives:** The aim of this study was to design a fully automated hybrid AI-based method, combining a convolutional neural network (CNN) and a tree-based model (XGBoost), which was capable of diagnosing different pelvic organ prolapses (POPs) in a dynamic two-dimensional ultrasound study from the midsagittal plane. **Methods:** This was a prospective observational study with 188 patients (99 with POP and 89 without POP). Transperineal pelvic floor ultrasound videos were performed, and normality or POP was defined. These videos were subsequently labeled, and an algorithm was designed to detect POP based on three phases: 1. Segmentation—a CNN was used to locate and identify the visible pelvic organs in each frame of the ultrasound video. The output had a very high dimensionality. 2. Feature engineering and dataset construction—new features related to the position and shape of the organs detected using the CNN were generated. 3. The POP predictive model—this was created from the dataset generated in the feature engineering phase. To evaluate diagnostic performance, accuracy, precision, recall, and F1-score were considered, along with the degree of agreement with the expert examiner. **Results:** The best agreements were observed in the diagnosis of cystocele and uterine prolapse (88.1%) and enterocoele (81.4%). The proposed methodology showed an accuracy of 96.43%, an overall accuracy of 98.31%, a recall of 100%, and an F1-score of 98.18% in detecting the presence of POP. However, when differentiating between the various types of POP, we observed that the precision, accuracy, recall, and F1-score were higher when detecting cystocele and uterine prolapse. **Conclusions:** We have developed the first predictive model capable of diagnosing POP in a dynamic, bi-dimensional ultrasound study from the midsagittal plane using deep learning and machine learning techniques.

## 1. Introduction

Pelvic organ prolapse (POP) is an increasing health problem that affects a large number of women, disrupting their quality of life [1]. Prevalence rates of POP have been reported to reach 50% [2]. Among women with POP, 6% are between the ages of 20 and 29, 31% are between 50 and 59 years old, and nearly 50% are 80 years old or older [3]. With the increase in life expectancy in future societies, the prevalence of POP is expected to rise, with an estimated increase to 46% by 2050 [4]. At present, the lifetime risk of a woman undergoing surgery for POP or stress urinary incontinence is between 11% and 20% [3,5,6], with recurrence rates of 30% after corrective POP surgery [5].

The pelvic organ prolapse quantification (POP-Q) system is considered the standard staging method for POP [7] and is the most widely used [8,9]. However, it has limitations, especially for the central compartment [10]. Additionally, when the POP-Q system is used preoperatively to stage POP, it has been noted to underdiagnose the severity of POP in the middle and posterior compartments [11]. These pre-surgical limitations of the POP-Q system can be mitigated through the use of transperineal ultrasound, which shows greater agreement than preoperative clinical examination in detecting POP in the central compartment with surgical indications [12]. Transperineal ultrasound has a sensitivity ranging from 60% to 93% and specificity between 64% and 95% [13,14,15,16,17] for diagnosing POP. However, ultrasound requires expertise from the examiner, who must perform manual measurements, leading to interobserver variability [18].

Currently, to automate pelvic floor ultrasound, artificial intelligence (AI) approaches are being used for the identification of different pelvic floor structures [19,20,21,22]. Interest in applying AI in the field of gynecology is increasing progressively, and it is expected to bring about a shift in vision within current medicine [23]. Machine learning (ML) is a field of AI that enables computers to learn to perform specific tasks based on experience, similarly to how humans learn. Within ML, deep learning (DL) involves the development of complex, specialized artificial neural networks for processing unstructured data, such as audio, images, or video. In this context, convolutional neural networks (CNNs) are one of the most widely applied DL techniques in medical image analysis. Specifically, they have been applied to static ultrasound studies of pelvic floor muscles [19,20,21,22] and for the dynamic ultrasound identification of various pelvic organs [24]. However, unlike static image studies, dynamic ultrasound is necessary for diagnosing pelvic floor dysfunctions such as POP [14,16], enabling a multicompartmental analysis during Valsalva maneuvers.

Dynamic ultrasound studies involve working with videos, meaning sequences with large numbers of images, which considerably increase the dimensionality of the problem and, with it, the risk of overfitting. A typical approach involves the expert manually selecting a few representative images from the video to directly train a CNN. However, this approach has the disadvantage of not being fully automatic, requiring expert intervention for the initial image selection. Our hypothesis is that we can work with full videos. To achieve this, we propose a two-phase approach: in the first phase, a CNN [24] is created to detect the position of different organs in each frame of the video; meanwhile, in the second phase, the information about the organs’ positions throughout the image sequence is used to train a tree-based model that predicts the existence of any POP. Based on the described approach, the goal of this work is to design a fully automated AI-based solution that allows for the diagnosis of different POP types in a dynamic two-dimensional ultrasound study from the midsagittal plane.

## 2. Materials and Methods

An observational and prospective study was designed with 188 patients. The patients were consecutively recruited from a specialized pelvic floor dysfunction clinic and a general gynecology clinic from 1 April 2023 to 31 September 2024.

This study was conducted in accordance with the Declaration of Helsinki and approved by Andalucia’s Board of Biomedicine Ethics Committee, with the code 0625-N-23. Informed consent was obtained from all subjects involved in this study.

### 2.1. Patient Inclusion

Two distinct patient groups were included: the first group consisted of patients with no pelvic floor pathology and the second group consisted of patients with symptomatic POP with surgical indications (symptomatic POP at stage 2 or greater and the failure of previous conservative treatment). The exclusion criteria included patients with a history of pelvic floor dysfunction, patients who had undergone prior corrective surgery for pelvic floor dysfunction, and patients with difficulty performing the Valsalva maneuver correctly. All patients were gynecologically evaluated before being included in this study and classified based on the presence or absence of POP. The clinical examination to assess POP was conducted by a pelvic floor dysfunction specialist using the POP-Q system [25], which indicated the need for the surgical correction of the POP. The following clinical parameters were included in the analysis: age, weight, height, body mass index (BMI), parity, menopausal status, and age at menopause.

### 2.2. Ultrasound Examination

All ultrasound examinations were performed by a single pelvic floor ultrasound expert (J.A.G.M.), who was blinded to the clinical data of the patients. A Toshiba^®^ 700 Aplio (Toshiba Medical Systems Corp., Tokyo, Japan) with a three-dimensional abdominal probe (PVT-675 MV) covered with a sterile glove was used for the ultrasound study. The transperineal pelvic floor ultrasound was performed with the patient in the dorsal lithotomy position. The transducer was carefully placed on the perineum, applying minimal pressure. For each patient, after confirming the correct performance of the Valsalva maneuver, a video was captured during the Valsalva maneuver (at least 6 s in duration) from rest. The diagnostic criteria for the different POPs in each compartment were as follows: Cystocele—urinary bladder descending 10 mm or more during Valsalva in relation to the posterior inferior edge of the pubic symphysis [14,26]. Uterine prolapse—a difference of 15 mm or more between rest and Valsalva in the pubis–uterine fundus distance [13,16,17]. Cervical elongation without uterine prolapse—a difference of less than 15 mm between rest and Valsalva in the pubis–uterine fundus distance, with the cervix descending 15 mm or more during Valsalva in relation to the posterior inferior edge of the pubic symphysis [13,16,17]. Rectocele—rectum descending 15 mm or more during Valsalva in relation to the posterior inferior edge of the pubic symphysis [14,26], with the herniation of the anterior rectal wall into the vagina [27]. Enterocele—enterocele region descending 15 mm or more during Valsalva in relation to the posterior inferior edge of the pubic symphysis [14,26], with abdominal content anterior to the anorectal angle, separating the vagina from the rectal ampulla [27].

A total of 188 videos were obtained (89 videos from patients without pelvic floor dysfunction and 99 videos from patients with POP).

### 2.3. Algorithm

**Figure 1:**
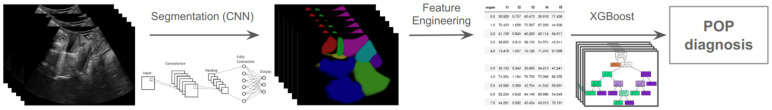
Workflow of proposed methodology.

A hybrid methodology combining a convolutional neural network (CNN) and a tree-based model from the gradient boosting machine (GBM) family [28] was developed to diagnose different POP types from dynamic two-dimensional ultrasound videos of the midsagittal plane. The proposed methodology can be broken down into three phases (see Figure 1). The following details each of the three phases of the methodology.

### 2.4. Segmentation

A CNN was used to locate and identify the visible pelvic organs in each frame of the ultrasound videos. The output had a very high dimensionality.

**Figure 2:**
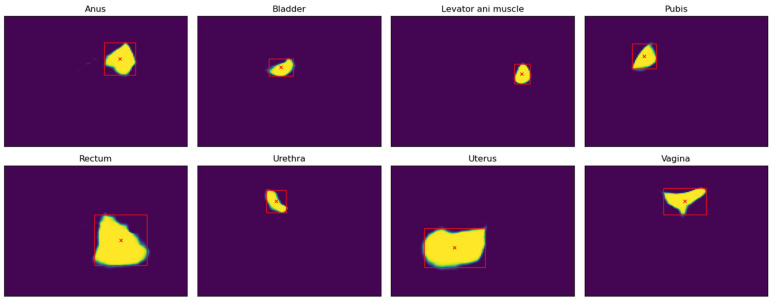
The CNN predictions for an ultrasound video. A confidence matrix was obtained for each of the organs visible in the frame.

The process began by extracting the sequence of frames from the ultrasound video, on which a CNN was used to locate (segment) the pelvic organs present in each frame of the video. As a result of this process, for each frame of the video, the position and shape of each visible organ were obtained, as shown in Figure 2. Specifically, for each organ and frame of the video, a confidence matrix (with values between 0 and 1) was generated, indicating, for each pixel of the original frame, the certainty with which the CNN predicted that the pixel belonged to a certain organ in a specific frame.

**Figure 3:**
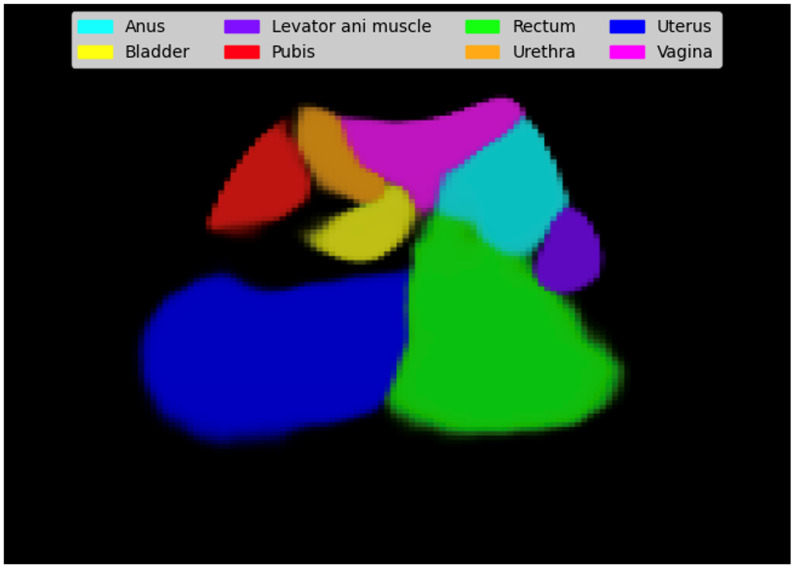
The result of the segmentation process of an ultrasound video frame using the CNN.

The methodology for building the pelvic organ segmentation CNN from ultrasound videos of the midsagittal plane was presented and validated in a previous study (see [24] for more details). It is important to note that the CNN used in the proposed methodology was trained on ultrasound videos from a set of patients different from the one used in the present study. The CNN was trained with videos of healthy patients, where the pelvic organs were manually labeled by an expert scanner (J.A.G.M.). The eight pelvic organs considered in this study were the pubis, urethra, bladder, vagina, uterus, anus, rectum, and levator ani muscle. Figure 3 shows the results of composing the confidence matrices represented in Figure 2.

It should be noted that the CNN was trained solely with videos from healthy patients; however, in this study, it was used to identify organs in patients with pelvic floor pathology. The idea behind this approach was the detection of anomalies. It was expected that the CNN would assign lower confidence values to organs that exhibited an unusual shape or were located in a different position than usual. Although the CNN would locate the organs in patients with pelvic floor pathologies with lower precision, this information would still be useful for the POP predictive model.

### 2.5. Feature Engineering and Dataset Construction

From the output of the CNN, new features related to the position and shape of the organs detected with the CNN were generated. As a result of this process, a data table was obtained, with one row for each sequence of frames and the columns representing the new generated variables.

**Figure 4:**
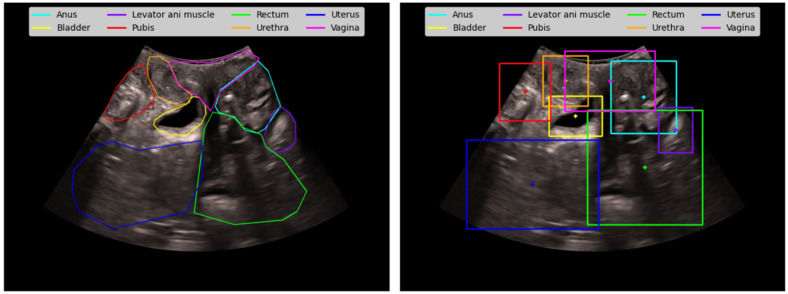
Contours (**left**) and bounding boxes and centroid (**right**) used in feature generation.

Once the CNN outputs (confidence levels at the pixel level) had been obtained for each frame of the video and each of the 8 possible organs, aggregated feature extraction was performed. First, variables were calculated at the frame and organ level, which were then aggregated at the sequence of frames and organ level. To achieve this, features related to the position and shape of the organs detected with the CNN, as well as the confidence (confidence level) with which they have been detected, were defined. These features can be divided into two categories:

- Confidence-based features: Basic statistical measures (mean, std, max, min) were calculated for the confidence values of the pixels in a frame given by the CNN for an organ. This same calculation was repeated considering only the pixels in the region of the frame where the CNN located the organ. This region was defined by the subset of pixels with a confidence greater than 0.5.
- Position- and shape-based features: In the calculation of these features, only the region of the frame where the organ has been located was considered. Once the organ’s region had been isolated, its centroid and the area, width, and height of the bounding box were calculated, as well as other features related to the organ’s coordinates in the frame. Figure 4 shows both the regions of the organs and their bounding boxes and centroids, which were considered for the calculation of these features.

Once these variables were calculated at the frame level, they were aggregated at the frame sequence level, calculating basic statistics (mean, std, max, min) over the sequence of values for each of these variables throughout the video. A total of 68 variables were defined to characterize the position and confidence with which the CNN identified an organ in a sequence of frames (which could be a full video or a segment of it). As a result of this aggregation process, a data record was generated with 68 variables for each organ and sequence of frames.

The second group of variables may seem the most interesting at first, as it characterizes the position of the organ, aiding in the detection of organs that deviate from their natural position. However, the confidence-based variables also play an important role, as mentioned earlier, since a decrease in the confidence values with which an organ has been detected would indicate some type of anomaly in it.

### 2.6. Data Augmentation

So far, the generation of an aggregated features record for each video and organ has been discussed. However, to increase the training data volume and reduce overfitting, several sub-sequences of frames were defined for each video. Specifically, to extract sub-sequences of frames from the complete videos, a sliding window scheme with N = 60 frames and an overlap of N/2 frames between sequences was used.

**Figure 5:**
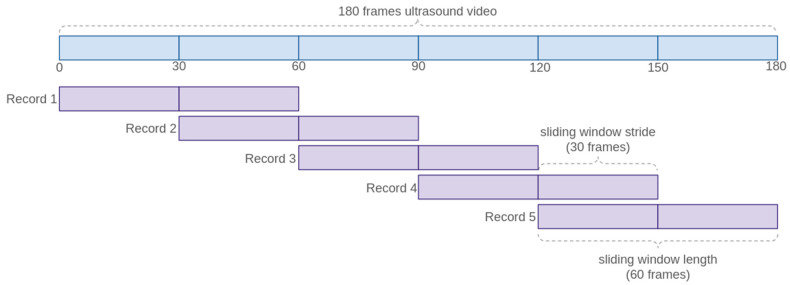
Sliding window with overlapping scheme used to augment and homogenize dataset.

As illustrated in Figure 5, for a video of 180 frames, 5 sub-sequences of 60 frames were generated. Additionally, as videos may have different durations, this approach standardized the length of the sequences in the training set.

### 2.7. Final Dataset Structure

For this study, 188 ultrasound videos were available (89 corresponding to patients without pelvic floor dysfunction and 99 to patients with POP). After applying the sliding window process, 677 sequences of frames were obtained, with an average of 3.6 frames per video. Additionally, the CNN segmentation process generated a different output for each of the 8 possible organs. Taking all of this into account, once the new variables had been calculated, a dataset with a total of 5416 rows and 69 columns was obtained. In addition to the 68 variables, a column indicating the organ corresponding to each row’s information was included. From this dataset, predictive models for different pelvic floor pathologies were built and evaluated.

This feature engineering phase greatly reduced the dimensionality of the dataset, which facilitated the generalization ability of the models trained on it. This was especially important considering the limited number of available patients. It is worth noting that the raw data (videos) initially contained 128 × 128 × N_frames features and consisted of a sample of 188 instances. After the segmentation and feature engineering stages, we obtained a dataset with 69 features and 5416 instances.

### 2.8. POP Predictive Model

A POP predictive model was built using the dataset generated in the feature engineering phase, which contained aggregated information about the position and shape of the organs detected with the CNN throughout the sequence of frames.

We built a predictive model for different pelvic floor pathologies, using the dataset generated in the feature engineering phase. For this task, XGBoost (eXtreme Gradient Boosting) [29] was selected, which is an evolution of a GBM and one of the machine learning techniques that provides the best results in a wide variety of domains.

In the ultrasound videos, different pelvic floor pathologies could be detected. Specifically, for this study, the following six pathologies were considered: cervical elongation, cystocele, cystourethrocele, enterocele, rectocele, and uterine prolapse. Each of the 188 videos in the dataset was examined by an expert scanner (J.A.G.M.), who determined which pathologies were present in each video, if any (89 videos corresponded to healthy patients). Each of these six pathologies represented a target in the predictive modeling process. In this work, each of the target variables (pathologies) were treated independently, meaning that, for simplicity, interactions between pathologies have not been considered. An additional target variable, termed “any prolapse”, was defined to indicate that the patient had some pelvic floor pathology, regardless of its type.

To train the predictive models, the data were randomly split into training and testing sets with 68% and 32% of the cases, respectively. Specifically, the test set contained data from 59 patients which, after the data preparation described in the previous phase, became a set of 1736 rows. The training set contained data from 129 patients which, after data treatment, contained 3680 rows.

As mentioned, a different model was fitted using each type of prolapse as the target variable. Although the overall ratio between healthy and affected patients was balanced in the dataset, the proportion of positive cases for each type of prolapse was not. For some pathologies, such as enterocele and cystourethrocele, the target variable was highly imbalanced. To avoid bias in model learning toward the majority (negative) class, greater importance was given to the minority (positive) class during training. To achieve this, positive cases were weighted according to the ratio between the number of negative and positive cases; that is, PosWeight = |Neg|/|Pos|.

**Figure 6:**
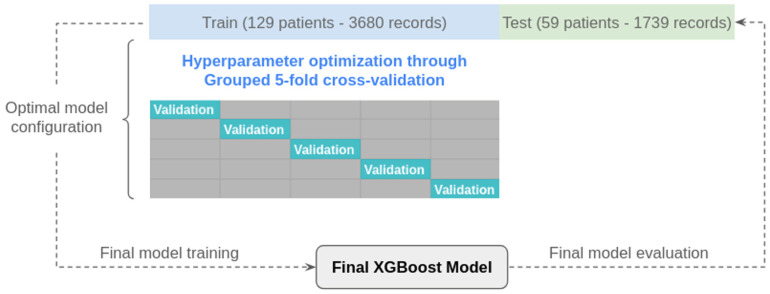
Validation scheme followed during hyperparameter optimization and model evaluation for one of the seven target variables.

A separate model was built for each of the seven possible target variables, following the scheme shown in Figure 6. First, hyperparameter optimization (randomized search) was performed for each model using the training set. To conduct a solid evaluation of each hyperparameter combination on the training set, a 5-fold cross-validation scheme was used. It is important to note that, after the feature engineering process, the dataset contained multiple records for each patient, which would lead to overfitting if a patient’s records were spread across more than one fold. To avoid this possible source of data leakage, a group-based scheme was used, known as grouped k-fold cross-validation, which ensured that all the records from a single case were in a single fold.

During hyperparameter optimization on the training set, the error was measured at the record level. The average precision (AP) metric was used for this, as it performs well in the case of imbalanced targets, such as in the case of rectocele or enterocele. AP summarizes a precision–recall curve as the weighted mean of precisions achieved at each threshold.

Once a final model had been obtained, it was evaluated on the test set. The models generated predictions at the record level (which represents information about a single organ extracted from sequences of 60 frames), and multiple records existed for each patient. To aggregate these predictions and obtain a final result at the patient level, the most frequent prediction was selected. Once predictions at the patient level had been obtained, various binary classification metrics were calculated to analyze the model’s predictions.

### 2.9. CNN Evaluation Metrics

To evaluate the diagnostic performance of the final model, based on the predictions at the patient level, on the test set, the following metrics were considered: accuracy, precision, recall, and F1-score [30,31], where accuracy = (TP + TN)/(TP + FP + TN + FN), precision = TP/(TP + FP), recall = TP/(TP + FN), and F1-score = 2/((1/Precision) + (1/Recall)), considering that TP = true positive, FP = false positive, TN = true negative, and FN = false negative. In addition, the degree of concordance between the predictions of the final model and the expert scanner’s findings was analyzed.

### 2.10. Computational Resources

The CNN used during the segmentation phase was trained with an NVIDIA GTX 1080Ti GPU (NVIDIA Corporation, Santa Clara, United States) installed alongside an Intel Core i5-7500 3.40 GHz CPU with Ubuntu 20 and 32 GB of RAM. To implement the networks, the Keras framework and the segmentation models package [14] were used.

Data processing and aggregation for the second stage and XGBoost model training and optimization in phase three were carried out on a dual Intel Xeon CPU E5-2660 v3 2.60 GHz running Debian 10 with 128 GB of RAM.

### 2.11. Statistical Analysis

The statistical analysis was performed using IBM SPSS Statistics version 26 (IBM, Armonk, NY, USA). The data were reviewed before the analysis. Numerical variables were described using mean values and standard deviations (SD) and, in the case of asymmetric distributions, medians and percentiles (p25 and p75) were used. Qualitative variables were expressed as percentages. Student’s *t*-test or the Mann–Whitney U-test was used for comparing numerical variables, and the χ^2^ test was used for qualitative variables.

## 3. Results

Of the 188 women included, 99 had some form of POP and 89 did not. Considering that different types of POP could coexist in the patients from this group, we had a total of 67 cystoceles, 13 cystourethroceles, 41 uterine prolapses, 36 cervical elongations, 9 enteroceles, and 24 rectoceles. The general characteristics of all the included patients are shown in Table 1. The average age was 57.1 ± 10.7 years, the average weight was 71.1 ± 12.0 kg, the average BMI was 27.0 ± 4.9, the average parity was 2.3 ± 1.1, the menopause status was present in 65.2% of patients, and the average age of menopause was 50.5 ± 3.4 years.

The videos corresponding to 129 patients were used to create the model. In this group, there were 74 patients with POP and 55 without. Among the patients with POP, considering that different types of POP could coexist in this group, there were 47 cystoceles, 6 cystourethroceles, 27 uterine prolapses, 27 cervical elongations, 8 enteroceles, and 18 rectoceles. The proposed methodology was validated with the videos from a group of 59 patients used to measure the diagnostic performance of the model. The validation group consisted of 27 patients with POP and 32 patients without POP. Among the patients with POP, considering that different types of POP could coexist in this group, there were 20 cystoceles, 7 cystourethroceles, 14 uterine prolapses, 9 cervical elongations, 1 enterocele, and 6 rectoceles.

Looking at the full set of all videos (Videos S1 and S2) from all the patients used to validate the model (Table 2), we see that the agreement with the expert examiner for detecting the presence of POP is 98.3%. However, these global results vary when focusing on specific types of POP. The best agreements are observed in the diagnosis of cystocele and uterine prolapse (both 88.1%) and enterocele (81.4%), with lower agreements for diagnosing cystourethrocele (71.2%), cervical elongation (67.8%), and rectocele (60.8%). The model presented a precision of 96.43%, an accuracy of 98.31%, a recall of 100%, and an F1-score of 98.18% for detecting the presence of POP. However, when differentiating the different types of POP, we observed that the precision, accuracy, recall, and F1-score were higher for detecting cystocele and uterine prolapse. The evaluation of the posterior compartment was more limited due to the small number of patients included.

## 4. Discussion

Upon validating the proposed methodology, we observed that it showed a high level of agreement with the expert examiner across the videos used for this purpose, with the model showing nearly 100% agreement (98.3%) for detecting the presence of any type of prolapse. Additionally, the model also showed good agreement with the expert examiner in diagnosing cystocele (88.1%), uterine prolapse (88.1%), and enterocele (81.4%), with more moderate agreements for cystourethrocele (71.2%), cervical elongation (67.8%), and rectocele (60.8%). Similarly, the precision, accuracy, recall, and F1-score yielded results between 96% and 100% in detecting the presence of any POP. When analyzing the different types of POP independently, the model performed better in precision, accuracy, recall, and F1-score when detecting cystocele, followed by uterine prolapse.

The approach proposed in this study is novel because it works with complete videos, and therefore does not require an expert to pre-select the frames for model training or predictions. However, working with complete videos (100–200 frames per video in this study) significantly increases the dimensionality of the problem. Training a CNN directly on a sequence of 100 frames, considering that we usually work with samples from several hundred patients, would lead to an overfitted model. In this sense, the first two steps (segmentation and feature engineering) of the developed methodology are critical, as they allow for a reduction in the dimensionality of the dataset from 128 × 128 × N_frames features to just a few dozen (69 in this case).

Additionally, the proposed methodology is transversal, in the sense that, with minor modifications, it can be applied to address other objectives related to ultrasound videos. Both the first part of the methodology—in which the regions of the frames where the different organs appear are identified—and the second part—where aggregated features related to organ identification and position are extracted—are generic and can be reused for other applications. One would only need to redefine the problem objective and adjust the new model as described in the third part of the methodology.

It could have been possible to train the CNN using videos from both healthy patients and patients with pelvic floor disorders, resulting in more accurate organ identification. However, this methodology would not be generalizable. The anomaly detection approach based on a CNN trained on healthy patients allows the methodology to be applied to other pathologies or objectives.

Different deep learning models applied to magnetic resonance imaging (MRI) images have been described for evaluating POP. One of the most recent is the model designed by Zhu et al. [32] which, using the vision transformer architecture and employing a label masking training strategy and pre-training methods, achieved a precision of 0.86 with a kappa coefficient of 0.77 [32]. In previous deep learning-based models for POP diagnosis using MRI, similar precision figures have been reported, such as in Wang X et al.’s study [33], which achieved a precision of 0.84. In fact, CNNs have been used to address the problem of blurred boundaries in soft tissues in pelvic MRI [34]. In a study using three-dimensional ultrasound for POP, it was observed that the application of a certain CNN model allowed for a diagnostic precision of 0.86 for POP [35]. In a recent study, where static ultrasound images at rest and during Valsalva were analyzed, recognition precisions greater than 91% were determined for different CNN-based models for identifying anterior compartment POP [36]. That work did not involve organ recognition, instead using heatmaps for prediction, which makes it more difficult to interpret [36]. However, to date, all of these studies have been based on the analysis of static POP images, either from MRI [32,33,34] or ultrasound [35,36]. The analysis of static images of the pelvic floor may occasionally be insufficient for an etiological diagnosis of POP, but a dynamic study is recommended to obtain the ultrasound diagnosis [14,16]. Furthermore, the study of a single compartment, as has been conducted in previous deep learning studies [36], does not allow one to analyze the multicompartmental relationship that exists between different pelvic organs during Valsalva maneuvers, which is a crucial aspect in POP diagnosis. Therefore, we wanted to consider these two premises in our study, developing a hybrid CNN-XGBoost methodology that allows for the dynamic ultrasound identification of POP while performing a multicompartment analysis. To date, this is the first study to have considered these two aspects in developing a model for the ultrasound identification of POP. AI-based ultrasound models are also being applied in other gynecological areas, such as the diagnosis of polycystic ovary syndrome. Di Michele et al. demonstrated the effectiveness of AI in improving the accuracy of ultrasound assessments of ovarian morphology and follicular count [37], further emphasizing the growing role of AI in gynecology.

### Strengths and Limitations

The main strength of this research is that it is the first published pilot study using a hybrid CNN-XGBoost methodology to diagnose dynamic, bi-dimensional ultrasound POP in a multicompartmental manner, detecting each organ independently, thus establishing the diagnostic basis for this methodology. Furthermore, the methodology used achieves very acceptable results without the need to dedicate considerable effort to labeling videos with POP. On the other hand, it must be considered that including videos, rather than static images, increases the difficulty for the model in performing its analysis, as the variability in videos for the same pathology is greater. Therefore, it is possible that our results are underestimated due to the number of patients included, which is our main weakness. Thus, we believe that by increasing the number of patients used to train both the CNN and the XGBoost diagnostic model in future studies, we will increase the diagnostic precision for POP. Another issue that could significantly improve model performance is the fact that each pathology was modeled independently. In other words, the developed model does not exploit the information of the coexistence of multiple pathologies in the same patient. In future studies, models should be considered that make predictions across all objectives simultaneously, such that interactions between them are considered.

Additionally, this study lacks external validation or comparison with examiners of different experience levels, which limits the generalizability of our findings. Furthermore, before clinical application, it is necessary to validate the model on a larger, multicenter cohort to ensure its robustness across different populations and settings. Another critical aspect to address in future work is a comprehensive error analysis, particularly in the posterior compartment, where a lower diagnostic performance was observed. Such an analysis would help to clarify the specific scenarios in which the model tends to fail and guide targeted improvements in model architecture or training data.

## 5. Conclusions

In conclusion, we developed the first predictive model capable of diagnosing POP in a dynamic, bi-dimensional ultrasound study from the midsagittal plane using deep learning and machine learning techniques.

## Figures and Tables

**Table 1:** The general characteristics of the patients included.

	Patients Included (n: 188)	
	Mean (±SD)	95% CI
Age	57.1 ± 10.7	55.6; 58.7
Weight	71.1 ± 12.0	69.4; 72.9
Height	1.6 ± 0.1	1.6; 1.63
BMI	27.0 ± 4.9	26.3; 27.8
Parity	2.3 ± 1.1	2.1; 2.5
Menopause	120/184(65.2%)	28.1%; 71.73%
Menopause age	50.5 ± 3.4	49.9; 51.2

**Table 2:** Accuracy of CNN for diagnosing different types of POP.

	Agreement with EE (n: 59)					
	%	95% IC	Precision (%)	Accuracy (%)	Recall (%)	F1-Score (%)
Any prolapse	98.3% (58/59)	95.0%; 100%	96.43%	98.31%	100%	98.18%
Cystocele	88.1% (52/59)	79.8%; 96.4%	74.07%	88.14%	100%	85.11%
Cystourethrocele	71.2% (42/59)	59.6%; 82.7%	18.75%	71.19%	42.86%	26.09%
Uterine prolapse	88.1% (52/59)	79.8%; 96.4%	70.59%	88.14%	85.71%	77.42%
Cervical elongation	67.8% (40/59)	55.9%; 79.7%	29.17%	67.80%	77.78%	42.42%
Rectocele	76.3% (45/59)	65.4%; 87.2%	27.78%	76.27%	83.33%	41.67%
Enterocele	81.4% (48/59)	71.5%; 91.3%	0%	81.36%	0%	-

## Data Availability

The dataset is available on request from the authors.

## Supplementary Materials

The following supporting information can be downloaded at: https://www.mdpi.com/article/10.3390/jcm14113634/s1, Video S1: Shows the video used to validate the CNN in the non-POP group. Blue: pubis; red: urinary bladder; orange: urethra; pink: uterus; purple: vaginal canal; yellow: anal canal; white: rectum; green: levator ani muscle; Video S2: Shows the video used to validate the CNN in the POP group diagnosed with cystocele and cervical elongation. Blue: pubis; red: urinary bladder; orange: urethra; pink: uterus; purple: vaginal canal; yellow: anal canal; white: rectum; green: levator ani muscle.

## List of Abbreviations

POP	Pelvic organ prolapse
POP-Q	Pelvic organ prolapse quantification
BMI	Body mass index
AI	artificial intelligence
CNNs	convolutional neural networks
GBM	Gradient boosting machine
XGBoost	eXtreme Gradient Boosting
TP	true positive
FP	false positive
TN	true negative
FN	false negative
